# A review of the injuries caused by occupational footwear

**DOI:** 10.1093/occmed/kqae003

**Published:** 2024-03-25

**Authors:** M C Pereira-Barriga, J M Borrero-Hernández, J J García-Iglesias, D López-López, C Ruiz-Frutos, R Allande-Cussó, J Gómez-Salgado

**Affiliations:** Department of Sociology, Social Work, and Public Health, University of Huelva, 21007 Huelva, Spain; Department of Sociology, Social Work, and Public Health, University of Huelva, 21007 Huelva, Spain; Department of Sociology, Social Work, and Public Health, University of Huelva, 21007 Huelva, Spain; Research, Health, and Podiatry Group, Department of Health Sciences, Faculty of Nursing and Podiatry, Industrial Campus of Ferrol, Universidade da Coruña, 15403 Ferrol, Spain; Department of Sociology, Social Work, and Public Health, University of Huelva, 21007 Huelva, Spain; Safety and Health Postgraduate Programme, Universidad Espíritu Santo, 092301 Guayaquil, Ecuador; Department of Nursing, University of Seville, 41009 Seville, Spain; Department of Sociology, Social Work, and Public Health, University of Huelva, 21007 Huelva, Spain; Safety and Health Postgraduate Programme, Universidad Espíritu Santo, 092301 Guayaquil, Ecuador

## Abstract

**Background:**

Occupational footwear is intended to provide protection against the risks associated with work activities. The choice of footwear is complex due to the welfare, health and safety conditions of workers.

**Aims:**

To identify the injuries and problems caused by occupational footwear through a systematic review of the existing literature.

**Methods:**

A literature search was carried out in the Cumulative Index to Nursing and Allied Health Literature, Dialnet Plus, Pubmed, Scientific Electronic Library Online, Medline, Scopus and Web of Science databases over the period 2000–23, following the PRISMA Declaration guidelines.

**Results:**

A total of 27 studies were included in the review. The results indicated that there is a wide variety of injuries caused by occupational footwear: from dermal injuries (e.g. calluses) and injuries to the nail apparatus to inflammatory pathologies such as plantar fasciitis or bursitis. In addition, inappropriate footwear can cause pain in the ankle and foot, knees, hips and lower back. Other results include the discomfort derived from the footwear itself.

**Conclusions:**

Inappropriate footwear can cause injuries to the foot and other related bone structures. Further studies are needed on the detection of foot injuries caused by occupational footwear and the levels of action at this level to improve the worker’s health, the adaptability of the footwear to the wearer, and the worker’s comfort and adherence to the footwear.

## Teaser

Occupational footwear provides protection to the foot, and its specifications must consider the specific characteristics of the feet (their morphology) and the biomechanics of work, as well as the environmental risks associated with the job. Workers report that their occupational footwear was uncomfortable, heavy, hot and did not fit their feet. Most of the authors attributed this problem to a lack of training in occupational risk prevention and adaptation to the specific needs of the wearer.

**Key learning points**

**What is already known about this subject**

- The specifications of professional footwear must consider the specific characteristics of the feet (their morphology) and the biomechanics of work.
- Identifying the injuries and pathologies that can occur in the foot due to the use of occupational footwear or footwear for professional use and its suitability is of utmost interest to prevent these lesions.

**What this study adds**

- There are several general occupational footwear injuries and others that are specific to the footwear used by staff in particular occupations.
- Most of the authors attributed this problem to a lack of training in occupational risk prevention and adaptation to the specific needs of the wearer.

**What impact this may have on practice or policy**

- Inappropriate use of footwear or use of inappropriate footwear has a negative impact on the workers’ health, leading to accidents at work, abandonment of this equipment and sick leaves due to workplace lesions.
- Further study of its potential for improvement would have a major positive impact on occupational health and decrease the rate of abandonment of this important personal protection equipment.

## Introduction

Occupational footwear provides protection to the foot from undesirable external stimuli. To prevent injuries in the work environment, many workers are required to wear occupational footwear for approximately 8 hours, 5 days a week, which means that a variety of designs in this type of footwear are required to meet the standards demanded [1–3]. Certain occupations operate in particularly hazardous environments such as the construction industry, underground mining, law enforcement agencies, firefighters and military personnel. Due to these particularly hazardous environments, occupational footwear is the common component of all occupational personal protective equipment (PPE). Occupational footwear consists of boots, shoes and clogs that are made of a variety of materials suited to each job, and even their manufacturing may vary within occupations depending on the tasks to cover [3].

The choice of appropriate occupational footwear is complex due to the welfare, health and safety conditions of workers. The specifications of the footwear must consider the specific characteristics of the feet (their morphology), the number of actions and movements required to carry out a job (biomechanics), as well as the environmental conditions of the surroundings and the demands of the activity itself [4,5]. Appropriate occupational footwear can prevent the occurrence of pathologies and skin disorders [5]. Still, it seems to be primarily designed for occupational safety purposes rather than functionality or comfort [1].

The choice shall be made on the basis of the results of a risk assessment of the workstation and shall require, in any case, a thorough knowledge of the workstation and its environment, as well as of the anatomical and physiological conditions and the health state of the person [6]. The minimum requirements concerning the choice and use of occupational footwear are laid down in Directive 89/656/EEC of 30 November and Regulation (EU) 2016/425 of the European Parliament (replacing Directive 89/686/EEC) [7–9]. A potential disadvantage of this approach is the lack of functionality or convenience and the possibility of posing a risk to the safety of the worker [1–3]. A research on 321 Australian workers by Marr and Quine [10] revealed that occupational footwear caused incipient foot problems and adversely affected existing foot problems in 91% of workers. The reported problems were, among others, foot pain (49%) and callouses (33%). Other issues related to safety footwear mainly included excessive heat (65%), inflexible soles (52%), shoe weight (48%) and pressure exerted by the steel toe cap (47%) [2,10].

Although in the field of occupational risk prevention, PPE must be chosen according to the results of a risk assessment of the workplace, in the case of footwear such an assessment alone cannot be considered sufficient, as the worker also needs to be assessed. The reason for this is that occupational footwear must conform to the morphology of the worker’s foot, which is an individualized one (not all workers have the same morphology) [4]. Therefore, the choice of occupational footwear must also consider certain foot morphologies such as pes cavus, flat feet or foot width. Some of the manifestations indicated by workers may be related to narrow footwear, non-breathable footwear, or footwear that does not respect the pressure points of their feet, thus causing calluses or pathologies of the nail apparatus. Discomfort resulting from occupational footwear is a common situation that in extreme cases may even lead to the worker stopping wearing them because they cannot tolerate the specific footwear available [2]. The interaction between the worker’s feet and their work surface can influence essential requirements of foot movement and when this interaction is adversely affected (e.g. loss of ankle range of motion), a multitude of variables such as the individual’s balance and gait can be affected [1–3].

Given the potential impact of occupational footwear on a worker’s physical function, it is necessary to ensure that it is designed to mitigate injury, not to become its cause [3]. The area of occupational footwear is comprehensively addressed by a set of standards at the European level (safety footwear, protective footwear and occupational footwear) [7–9]. In this sense, regulation not only exists for the design of footwear, but also for the testing methods to check its suitability for the indicated workplace. With this premise in mind, when manufacturing footwear as PPE, another consideration should be borne in mind: the comfort the footwear offers [5,7–9].

Regarding this comfort in footwear, it refers to the adaptation of the footwear to the worker’s foot in such a way that it does not cause injury or discomfort, and that it respects the morphology of the individual’s foot in terms of its pressure points and physiological conditions. In other words, it must fit well enough so that the worker does not feel discomfort, and it must reduce the injuries caused by the PPE itself so that the worker does not have the desire to stop wearing it [1–3,5].

Therefore, identifying the injuries and pathologies that can occur in the foot due to the use of occupational footwear or footwear for professional use and its suitability is of utmost interest to prevent these lesions, as well as to avoid discontinuing the use of such footwear. Similarly, it is important to review current field studies to verify the existing scientific evidence. Thus the aim of the present review was to identify the injuries and pathologies caused by occupational footwear among the working population.

## Methods

Following the PRISMA Declaration guidelines [11], a systematic review of studies that investigated foot and lower limb injuries and pathologies related to occupational footwear and their incidence in various sectors of the labour market was carried out. The protocol followed is listed in the International Prospective Register of Systematic Reviews (PROSPERO) with code CRD42023428195. The PRISMA method was chosen for this study as it is the most widely accepted systematic review system as regards scientific evidence and, therefore, the most suitable for this type of study. No approval by an ethics committee was needed.

Using the PEO format (Population, Exposure, Results) [12], the research question from which the keywords used were to be derived was formulated (Table 1).

**Table 1.** Format: PEO

Population	Worker in any sector.
Exposure	Occupational footwear, safety footwear or prospective footwear.
Results	Injuries caused by footwear and their frequency, degree of discomfort, worker’s adherence to occupational footwear, appropriateness of occupational footwear worn, field/sector/context studied, type of study, participants.

The search was carried out in the following electronic databases: Cumulative Index to Nursing and Allied Health Literature, Dialnet Plus, Pubmed, Scientific Electronic Library Online, Medline, Scopus and Web of Science. The Medical Subject Headings (MeSH) descriptors used were Wounds and Injuries, Shoes and occupational groups, supplemented with related free terms such as injuries, work shoes, work, footwear, safety, safety footwear, professional footwear, occupational footwear and work footwear (Supplementary Material 1, available as Supplementary data at *Occupational Medicine* Online). To enlarge the scope of the search, synonymous terms were used based on the MeSH descriptors, linked by the Boolean operators AND and OR. The search strategy was carried out between 28 October 2022 and 6 May 2023 (Table 2).

**Table 2.** Search strategy used according to each database

Database	Search strategy	Results
CINAHL	work shoes AND injuries	319
CINAHL	footwear AND work AND injuries	38
Dialnet Plus	calzado de uso profesional	39
Dialnet Plus	calzado laboral	137
Scielo	calzado laboral	13
Scielo	calzado de uso profesional	2
Scielo	calzado de trabajo	58
Medline (Ebsco)	footwear AND work AND injuries	89
Medline (Ebsco)	safety footwear	53
CINAHL	safety footwear	50
Medline (Ovid)	footwear AND work AND injuries	48
Medline (Ovid)	safety footwear	16
Medline (Proquest)	footwear AND work AND injuries	132
Medline (Proquest)	safety footwear	239
Scopus	(TITLE-ABS-KEY (footwear) AND TITLE-ABS-KEY (work) AND TITLE-ABS-KEY (injuries))	142
Scopus	TITLE-ABS-KEY (safety AND footwear)	539
Web Of Science	(ALL = (work shoes)) AND ALL = (injuries)	389
Web Of Science	((ALL = (footwear)) AND ALL = (work)) AND ALL = (injuries)	248
Web Of Science	ALL = (safety footwear)	476
Pubmed	(footwear [All Fields] AND (‘work’[MeSH Terms] OR ‘work’[All Fields])) AND (‘injuries’[Subheading] OR ‘injuries’[All Fields] OR ‘wounds and injuries’[MeSH Terms] OR (‘wounds’[All Fields] AND ‘injuries’[All Fields]) OR ‘wounds and injuries’[All Fields]) AND (‘open access’[filter] AND medline[sb])	1219
Pubmed	((‘work’[MeSH Terms] OR ‘work’[All Fields]) AND (‘shoes’[MeSH Terms] OR ‘shoes’[All Fields])) AND (‘injuries’[Subheading] OR ‘injuries’[All Fields] OR ‘wounds and injuries’[MeSH Terms] OR (‘wounds’[All Fields] AND ‘injuries’[All Fields]) OR ‘wounds and injuries’[All Fields]) AND (‘open access’[filter] AND medline[sb])	2475
Pubmed	((‘safety’[MeSH Terms] OR ‘safety’[All Fields]) AND footwear [All Fields]) AND (‘open access’[filter] AND medline[sb])	1541
Pubmed	((Employee*[Title/Abstract] OR Personnel[Title/Abstract] OR Worker*[Title/Abstract] OR ‘Occupational Group’[Title/Abstract]) AND (shoe*[Title/Abstract] OR footwear[Title/Abstract] OR ‘safety footwear’[Title/Abstract] OR foot[Title/Abstract])) AND (‘Injuries and Wounds’[Title/Abstract] OR ‘Wounds and Injury’[Title/Abstract] OR ‘Injury and Wounds’[Title/Abstract] OR Trauma*[Title/Abstract] OR ‘Research-Related Injur*’[Title/Abstract] OR Injuries[Title/Abstract] OR Injury[Title/Abstract] OR Wound*[Title/Abstract]) Filters: from 2013 – 2023	209
Web of Science	‘Injuries and Wounds’ OR ‘Wounds and Injury’ OR ‘Injury and Wounds’ OR Trauma* OR ‘Research-Related Injur*’ OR Injuries OR Injury OR Wound* (Topic) AND shoe* OR footwear OR ‘safety footwear’ OR foot (Topic) AND Employee* OR Personnel OR Worker* OR ‘Occupational Group’ (Topic) and 2013 or 2014 or 2015 or 2016 or 2017 or 2018 or 2019 or 2020 or 2021 or 2022 or 2023 (Publication Years)	824
Date of search 06/05/2023	Total	9295

For inclusion criteria, admitted articles were written in English, Spanish, French or Portuguese, and were published between 2013 and 2023. Articles that assessed all types of safety footwear and its effects on the worker in any occupational sector, specifically injuries and potential discomfort, were included, as these met the objectives to be studied in this review. Articles reporting field studies and/or literature reviews that met the quality criteria of the Joanna Briggs Institute (JBI) at the University of Adelaide (Australia) [13] were also included. Articles that did not address the research question and those that could not be retrieved from databases were excluded.

Two researchers (M.C.P.B. and J.M.B.H.) independently searched and selected the included articles according to the established criteria, and then agreed on the results. Discrepancies were resolved by a third author (J.J.G.I.). The methodological quality of the selected studies was determined using the JBI critical appraisal tools for studies [13]. These tools are used to assess the methodological quality of a study and to determine the extent to which a study has excluded or minimized the possibility of bias in its design, conduct and/or analysis. The version for quantitative cross-sectional studies (8 items) was used [14], setting a cut-off point of 6 for acceptance for inclusion in this review (Supplementary Material 2, available as Supplementary data at *Occupational Medicine* Online), and for systematic reviews, setting a cut-off point of 9 (11 items) (Supplementary Material 3, available as Supplementary data at *Occupational Medicine* Online) [15].

## Results

**Figure 1.**
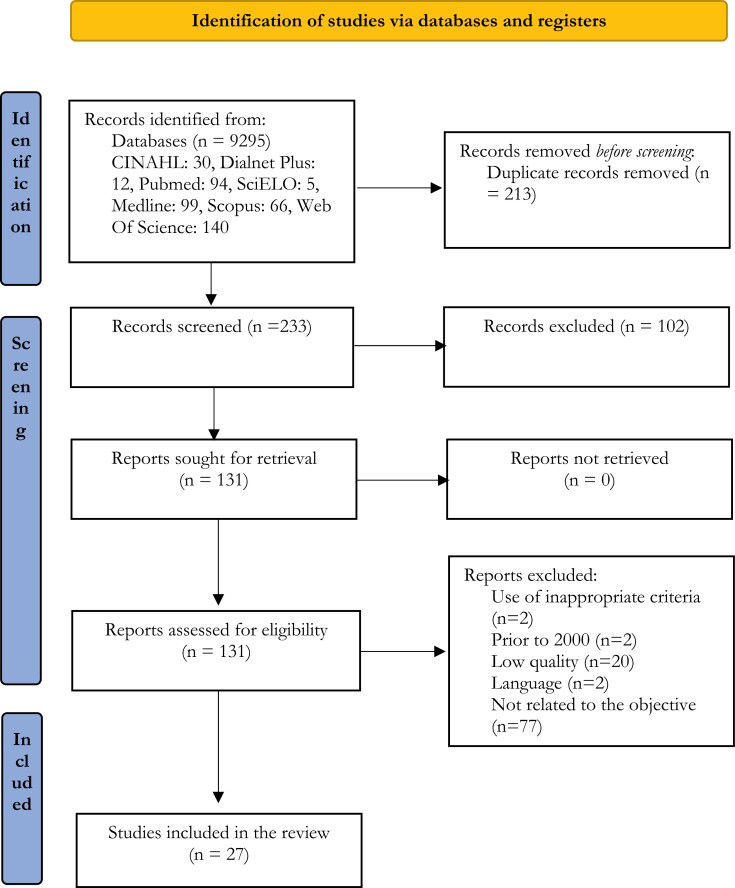
Search results (Flowchart—PRISMA) [11]

The initial search strategy identified a total of 9295 references, which were then screened according to the objective of this review. Finally, a total of 27 studies were selected [1–5,16–37], as shown in Figure 1, following the PRISMA statement [11].

Regarding the countries in which the studies were conducted, 3 of the 27 studies were conducted in Australia [24,25,35], another 3 in Spain [5,23,33], 3 in the USA [19,32,34], 1 in Colombia [18], 1 in China [31], 1 in Paraguay [17], 2 in Greece [27,36], 1 in Japan [21], 1 in Ethiopia [20], 2 in Germany [2,30], 2 in Iran [29,37], 1 in Bolivia [4] and 1 in Latvia [28].

Most of the studies (*n* = 5) were conducted in the setting of a hospital [17–21]. Others were conducted in miners (*n* = 2) [24,25], seafarers (*n* = 1) [5], the wine industry (*n* = 1) [35], farmers (n = 2) [34,37], aerobics instructors (*n* = 1) [36], firefighters (*n* = 2) [31,32], aircraft and automotive industry workers (*n* = 1) [2], and military personnel (*n* = 4) [27–30]. They were also carried out in industries with the support of universities (*n* = 1) [4] or researching different sectors at the same time (*n* = 4) [1,3,4,23,33]. There were also two bibliographic reviews in the nursing sector [16,22], two in different work sectors [3,23], one in military personnel [26], and another one describing the different types of occupational footwear, both for the civil and military sectors [1].

The results shown in the study by Maidana de Zarza *et al*. [17], in which a sample of 1037 nursing professionals was studied, showed that 52% of the subjects considered clogs without a heel pad to be comfortable, while 36% considered them to be dangerous. Moreover, 73% considered that unsuitable footwear could cause various pathologies such as sprains and muscle cramps, among others. Self-perceived danger can be considered a risk factor for resorting to other types of footwear of various kinds, in that appropriate footwear with the right anthropometric characteristics is essential for the daily demands of the job [18].

Nealy *et al*. [19] specified the most frequent types of problems that can be caused by inappropriate footwear such as plantar fasciitis, metatarsalgia, heel bursitis, bone spurs and Achilles tendonitis, with the main causes being inadequate self-care [17,19,20]. For their part, Tojo *et al*. [21] and Pedraza-Melo *et al*. [18] related the comfort associated with the footwear to both personal and psychological factors. In this line, Bernardes *et al*. [22] go further and conclude that interventions at this level are complex because of the context of the individual worker and the personal traits of the worker.

In other sectors such as the construction, telecommunications, and the hotel and catering industry, the same problems were observed, to which other problems could be added such as those derived from the use of closed footwear with reinforced toecaps, which results in greater weight and less comfort [23]. In addition, in sectors such as the wine industry, the aeronautical industry and firefighting, more than half of the workers had calluses, dry skin and tinea pedis, as well as pain in the foot, specifically in the sole, external malleolus, heel, calcaneus and cuboid areas [24,25]. Finally, in a group with a special regime, the military personnel, no difference was observed in terms of a higher incidence of injuries when using military boots [26–29], with the exception of the study by Schulze *et al*. [30], which concluded that footwear had a significant influence on gait parameters and the functionality of the lower limb. Supplementary Table S4 (available as Supplementary data at *Occupational Medicine* Online) shows the main characteristics and findings of the studies included in this literature review.

## Discussion

The aim of this study was to identify the injuries and problems caused by occupational footwear. In this case, several alterations have been found as a result of their use such as plantar fasciitis, metatarsalgia, heel bursitis, bone spurs and Achilles tendonitis, with the main causes being inadequate self-care [17,19,20]. Other authors [18,21] identified the problem at the level of comfort associated with the footwear as both personal and psychological factors, requiring an individual and personal approach to the worker to study them [22]. In fact, the same injuries are observed in jobs belonging to different sectors [23] with problems related to the physical and technical specificities of the footwear used for each workstation [24,25]. This could be explained by the fact that occupational footwear has a series of common characteristics (closed, with laces, with insoles, that support the weight of the worker, among others) and specific characteristics for each job (reinforced toecap, lightweight, puncture-proof soles, etc.)

On the other hand, podiatric problems, foot pain and the comfort of occupational footwear were not the only factors studied. Other factors such as foot temperature [31], impaired balance, gait and stability of the individual [2,32], performance with respect to the required tasks [3], and possible allergies to footwear materials [33] may be relevant when it comes to favouring injuries or decreasing comfort. Similarly, the context in which each job was carried out also had an influence. In fact, the study by Rivas-López *et al*. [5] concluded that offshore workers had a higher rate of fractures and need for pharmacological treatment than onshore workers. Along the same lines, a study carried out on 10 workers on a farm revealed other types of injuries such as crushing of the foot by large animals or snake bites to the worker’s foot [34].

Lastly, understanding the biomechanical and muscular functioning of the foot while wearing safety footwear can help to detect risk factors related to the occurrence of injuries [38,39]. In this sense, Richarson *et al*. [16] highlighted in their literature review that occupational footwear, especially if it is unstable, influences musculoskeletal injuries in the healthcare setting. Both the flexibility of the footwear (depending on the type of work to be carried out) and the use of insoles [40–43] or the microclimate inside the footwear [44] may be future elements of study, and the Industry 4.0, through the use of sensor systems connected to monitors that collect data in real time, could be the adequate approach [45]. In fact, until a few years ago, it was not permitted to modify the safety footwear to correct misalignments of the foot, nor was it permitted to tailor safety footwear to meet the specific needs of the wearer [7–9].

All the aforementioned findings have a negative impact on the health of the worker in the workplace, leading to accidents at work, abandonment of this PPE, sick leaves due to back, neck and knee pain originating in the muscles, and even ‘poor posture’ caused by the footwear itself [2,8]. Further study of the negative implications of this type of footwear and an assessment of its potential for improvement would have a major positive impact on occupational health, as well as on reducing sick leaves and accidents at work, and decreasing the rate of abandonment of this important PPE by minimizing the negative consequences of its use [22].

This review has several limitations. First, certain studies were rejected for language reasons (languages not listed in the inclusion criteria due to poor language proficiency, thus impeding full understanding) and other articles that met the requirements for inclusion may have been left out. Similarly, studies that could not be accessed through the database because the institution had not subscribed and were therefore not accessible for consultation were also rejected. Second, the resulting representative sample was small, so studies prior to the chosen period (2013–23) that met the requirements for inclusion should have been included to increase the sample size. Moreover, this limitation could be solved in the future with further studies on this topic. Third, not all authors used the same measurement instruments or the same variables, so the results obtained could not be homogeneous. Lastly, not all studies were of equal quality according to the JBI, which also affected the homogeneity of the results.

In summary, the most common injuries caused by occupational footwear are lower back pain, hip pain, knee pain and foot pain. Nail pathologies, calluses, dry skin, tinea pedis and allergic reactions can also be found among other dermal foot pathologies. Other pathologies such as muscle cramps, plantar fasciitis, metatarsalgia, heel bursitis, bone spurs, hammertoes or Achilles tendonitis should also be mentioned.

Workers reported that their occupational footwear was uncomfortable, heavy, hot and did not fit their feet. Most of the authors attributed this problem to the lack of training in occupational risk prevention for the worker when choosing suitable footwear, but action is also considered at the level of the choice of occupational footwear for the worker.

Literature on occupational footwear focuses on the risk of falls at the same level by improving slip-resistant soles of the footwear, but much more research is needed along the lines of the injuries described above to improve occupational footwear. This improvement will be easier to achieve, thanks to the adaptation of European regulations in the near future.

In short, further studies should be carried out on the detection of foot injuries due to occupational footwear and the levels of action at this level to improve the health of the worker, the adaptation of occupational footwear to the user, its comfort and the adherence of the worker to the footwear.

## Supplementary Material

kqae003_suppl_Supplementary_Tables_S1-S3

kqae003_suppl_Supplementary_Tables_S4
