# Supplementary material for: A review of the injuries caused by occupational footwear
Source: Occup Med (Lond). 2024 Mar 25;74(3):218–24. doi: 10.1093/occmed/kqae003 (PMC11080658; doi:10.1093/occmed/kqae003)
Supplement: kqae003_suppl_Supplementary_Tables_S1-S3 [file kqae003_suppl_supplementary_tables_s1-s3.doc]

**SUPPLEMENTARY MATERIAL**

**Table S1. Definitions and search terms**

| **Terms** | **Definition** | **Search terms** |
| --- | --- | --- |
| Wounds and Injuries | Damage inflicted on the body as the direct or indirect result of an external force, with or without disruption of structural continuity. | “Injuries and Wounds” OR “Wounds and Injury” OR “Injury and Wounds” OR Trauma* OR “Research-Related Injur*” OR Injuries OR Injury OR Wound* |
| Shoes | Footwear made of a flat sole shaped to the contour of the foot, connected to an upper portion which may or may not completely cover or wrap around the foot. | shoe* OR footwear OR “safety footwear” OR foot |
| Occupational Groups | Members of the various professions (e.g., PHYSICIANS) or occupations (e.g., POLICE). | Employee* OR Personnel OR Worker* OR “Occupational Group” |

| **Table S2. Scores of cross-sectional observational studies** | | | | | | | | | |
| --- | --- | --- | --- | --- | --- | --- | --- | --- | --- |
| **Studies** | **JBI** | **The participants and the environment are described in detail** | **Inclusion criteria are clearly defined** | **Exposure was measured in a valid and reliable way** | **The criterion used to measure the condition was objective** | **Confounding factors were identified** | **Strategies for dealing with confounding factors** | **Results measured in a valid and reliable way** | **An appropriate statistical analysis was used** |
| Dobson et al. (24) | 7/8 | Yes | Yes | Yes | No | Yes | No | Yes | Yes |
| Dobson et al. (25) | 6/8 | Yes | Yes | Yes | No | Yes | No | Yes | Yes |
| Rivas-López et al. (5) | 7/8 | Yes | Yes | Yes | Yes | Yes | No | Yes | Yes |
| Tian et al. (31) | 8/8 | Yes | Yes | Yes | Yes | Yes | Yes | No | Yes |
| Maidana de Zarza et al. (17) | 6/8 | Yes | Yes | No | No | Yes | Yes | Yes | Yes |
| Gatica-Ortega et al. (33) | 8/8 | Yes | Yes | Yes | Yes | Yes | Yes | Yes | Yes |
| Cooper et al. (35) | 6/8 | Yes | Yes | Yes | No | Yes | No | Yes | Yes |
| Nealy et al. (19) | 7/8 | Yes | Yes | Yes | No | Yes | Yes | Yes | Yes |
| Neil (34) | 6/8 | Yes | Yes | Yes | Yes | No | No | Yes | Yes |
| Sáenz (4) | 6/8 | Yes | Yes | Yes | No | Yes | No | Yes | Yes |
| Pedraza-Melo et al. (18) | 6/8 | Yes | Yes | Yes | Yes | No | No | Yes | Yes |
| Mencia Fernández (23) | 6/8 | Yes | Yes | Yes | No | Yes | No | Yes | Yes |
| Malliou et al. (36) | 6/8 | Yes | Yes | Yes | Yes | No | No | Yes | Yes |
| Tojo et al. (21) | 6/8 | Yes | Yes | Yes | No | Yes | No | Yes | Yes |
| Getie et al. (20) | 6/8 | Yes | Yes | Yes | Yes | No | No | Yes | Yes |
| Ochsmann et al. (2) | 8/8 | Yes | Yes | Yes | Yes | Yes | Yes | Yes | Yes |
| Sharifirad et al. (37) | 6/8 | Yes | Yes | Yes | No | Yes | No | Yes | Yes |
| Schulze et al. (30) | 7/8 | Yes | Yes | Yes | Yes | Yes | No | Yes | Yes |
| Sobhani et al. (29) | 6/8 | Yes | Yes | Yes | Yes | No | No | Yes | Yes |
| Paisis et al. (27) | 6/8 | Yes | Yes | Yes | Yes | No | No | Yes | Yes |
| Nesterovica et al. (28) | 8/8 | Yes | Yes | Yes | Yes | Yes | Yes | Yes | Yes |
| Garner et al. (32) | 6/8 | Yes | Yes | Yes | Yes | No | No | Yes | Yes |

| **Table S3. Scores of systematic reviews** | | | | | | | | | | | | |
| --- | --- | --- | --- | --- | --- | --- | --- | --- | --- | --- | --- | --- |
| **Studies** | **JBI** | **The review question is clearly and explicitly formulated** | **The inclusion criteria were appropriate for the review question** | **The search strategy was adequate** | **The sources and resources used for the search for studies were adequate** | **The study assessment criteria were adequate** | **The critical assessment was carried out by two or more reviewers independently** | **There were methods to minimise errors in data extraction** | **The methods used to combine the studies were appropriate** | **The likelihood of publication bias was assessed** | **Policy and/or practice recommendations were supported by the data reported** | **Specific directives for further research were appropriate** |
| Bernardes et al. (22) | 10/11 | Yes | Yes | Yes | Yes | Yes | Yes | Yes | Yes | No | Yes | Yes |
| Dobson J.A. et al. (1) | 10/11 | Yes | Yes | Yes | Yes | Yes | Yes | Yes | Yes | No | Yes | Yes |
| Richardson et al. (16) | 11/11 | Yes | Yes | Yes | Yes | Yes | Yes | Yes | Yes | Yes | Yes | Yes |
| Orr et al. (3) | 10/11 | Yes | Yes | Yes | Yes | Yes | Yes | Yes | Yes | No | Yes | Yes |
| Wardle & Greeves (26) | 10/11 | Yes | Yes | Yes | Yes | Yes | Yes | Yes | Yes | No | Yes | Yes |
