# Supplementary material for: A review of the injuries caused by occupational footwear
Source: Occup Med (Lond). 2024 Mar 25;74(3):218–24. doi: 10.1093/occmed/kqae003 (PMC11080658; doi:10.1093/occmed/kqae003)
Supplement: kqae003_suppl_Supplementary_Tables_S4 [file kqae003_suppl_supplementary_tables_s4.docx]

| **Suppl_Table 4. Characteristics of the studies included in the systematic review** | | | | | |
| --- | --- | --- | --- | --- | --- |
| **Study** | **Context** | **Type of study** | **Methods** | **Participants** | **Main findings** |
| Dobson et al. (24) | Illawara Coal employees at Dendobrium and West Cliff (Australia) - 2016 | Descriptive cross-sectional study | Questionnaire with 54 questions: Measuring miners' satisfaction with their work boots in relation to their requirements | 358 underground miners | Low back pain, hip pain, knee pain, ankle pain, and foot pain. Foot problems: hallux abducto valgus (HAV), pain in the 1^st^ metatarsophalangeal joint (MFA), pain in the inner arch of the foot (MLA), hyperkeratosis, sole pain, pain in the lateral malleolus area, pain in the heel, pain in the calcaneal area, pain in the cuboid area. |
| Dobson et al. (25) | Illawara Coal employees at Dendobrium and West Cliff (Australia) - 2016 | Descriptive cross-sectional study | Questionnaire with 54 questions: Measuring miners' satisfaction with their work boots in relation to their requirements | 358 underground miners | Foot problems reported by 55% of participants: calluses (33%), dry skin (30%), tinea pedis (13%). They also reported lower back pain (45%), foot pain (42%), knee pain (22%), and ankle pain (25%). |
| Rivas-López et al. (5) | Galician Sea Workers (Spain) (Sep 2013 to June 2014) | Descriptive cross-sectional study | Socio-demographic variables and podiatric pathologies | Seafarers (n=47) and shore-based workers (n=47) residing in Galicia | Seafarers have a higher rate of fractures than land-based workers, a higher rate of drug treatment, and a higher rate of podiatric pathology. |
| Tian et al. (31) | China - 2022 | Descriptive cross-sectional study | 4 conditions: with/without training shoes and with/without firefighting boots (standing and walking) | 13 healthy male volunteer firefighters | The results showed that wearing firefighting boots may elevate the thermal sensation of wearers. The PPE load increased the in-shoe and foot skin temperature. |
| Maidana de Zarza et al. (17) | Clinical Hospital, San Lorenzo (Paraguay) - 2017 | Descriptive cross-sectional study | Footwear suitability and self-care variables | 1037 nursing professionals of the Clinical Hospital | 52% considered clogs without heel pads comfortable, 36% dangerous; 74% considered that inappropriate footwear during working hours could lead to ankle sprains, claw toes, malleolar oedema, and muscle cramps; 65% said that the ideal footwear for occupational use is footwear that adapts to the movements of the feet in a way that makes them move efficiently; 28% indicated that it should adequately protect the tarsal and metatarsal joints. |
| Gatica-Ortega et al. (33) | Forklift worker (Spain, 2021) | Descriptive cross-sectional study | Clinical case study | 52-year-old man who presented with spontaneous urticaria with a 5-day course | Severe symmetrical erythema with oedema and coalescing blisters present. The lesions were present on the areas of the foot that were in contact with a leather safety shoe. He also suffered from fever and arthralgia in the ankle and wrists. |
| Cooper et al. (35) | Australian wine industry (2021) | Descriptive cross-sectional study | 82-question survey | 207 wine industry workers | Foot pain in 37% of participants, ankle pain (18%), toe pain (13%), heel pain (11%). 18% were treated by podiatrists. One third responded that their work shoes made their feet hurt and more than half reported that their boots were heavy and hot. |
| Nealy et al. (19) | Hospital (USA, 2012) | Descriptive cross-sectional study | Survey conducted with online tool | 2000 female nurses | Problems such as plantar fasciitis, metatarsalgia, heel bursitis and bone spur were reported, as well as hammertoes, Achilles tendonitis, high arches, flat feet, and bunions. |
| Neil (34) | Southeastern US farm  (2002) | Descriptive cross-sectional study | Wounds, pathologies, and conditions variables | 100 farmers | 83% suffered blisters on feet or legs due to shoes or boots, 73% had had their foot stepped on by a large farm animal, 7% were bitten by a snake (venomous or not). Of the remaining ones: 22% foot or leg injury due to a fall. |
| Sáenz (4) | Kóndor Footwear Company (Bolivia, 2006-2007) | Descriptive cross-sectional study | Methodological criteria study | 143 workers | The most common diseases in both men and women were fungal infections, bunions, and ingrown toenails. Calluses occurred with increasing age. |
| Pedraza-Melo et al. (18) | Hospital (Colombia, 2012) | Descriptive cross-sectional study | Footwear characteristics, physical examination of the foot, and assessment of the work area | 60 nurses | Pain in the lower heel area (15%) and sole of the foot (12%). Signs and symptoms detected were difficulty running, and swollen feet and ankles. Helomas (2%), dry skin (10%), onychomycosis (7%). |
| Mencia Fernández (23) | Different sectors (Spain, 2008-2009) | Bibliographic review + Descriptive cross-sectional study | Variables: types of footwear, time of use, pathologies, suitability, etc. | 63 workers | Thickening of the nail plate (2%), ingrown toenail (17%), nail ridges (2%), presence of calluses and helomas (27%), dry skin (18%), heel ridges (10%), changes in nail curvature (3%), changes in nail colouring (8%), flat feet (5%), pronated feet (5%), pes cavus (3%). |
| Malliou et al. (36) | Aerobics instructors (Greece, 2012) | Descriptive cross-sectional study | Number of injuries requiring medical care | 273 aerobics instructors | The main factors for injury were inappropriate footwear, long hours, and different types of dancing and floor condition. |
| Tojo et al. (21) | Hospital (Japan, 2017) | Descriptive cross-sectional study | Standardized Nordic Questionnaire and the Manchester Foot Pain and Disability Index | 640 nurses | Foot and ankle pain or discomfort (23%) associated with uncomfortable footwear worn at work. |
| Getie et al. (20) | Hospital (Ethiopia, 2021) | Descriptive cross-sectional study | Standardized Nordic Questionnaire | 366 nurses | Ankle and foot pain (44%) located in: heel (14%), big toe (13%), ankle (11%), plantar forefoot (11%), medial arch (9%), lesser toes (9%). |
| Bernardes et al. (22) | Healthcare setting | Systematic literature review | Systematic literature search following the PRISMA statement | Articles from different databases | Differences in the way of defining foot disorders, combined with the multiple compound working contexts and personal traits, generate a great variability of podiatric clinical prevalence rates, thus increasing complexity when interventions are needed at an occupational level. |
| Dobson J.A. et al. (1) | Civilian or military population | Systematic literature review | Systematic literature search following the PRISMA statement | 18 articles resulting from the literature search | There is limited research on the influence of the work boots on the worker's gait. |
| Richardson et al. (16) | Musculoskeletal injuries in the nursing profession | Systematic literature review | Systematic literature search following the PRISMA statement | 20 articles resulting from the literature search | The literature is scarce. Occupational footwear, especially if unstable, influences musculoskeletal injuries in the nursing profession. |
| Ochsmann et al. (2) | Automotive industry (Germany, 2016) | Descriptive cross-sectional study | Study of plantar pressure in subjects wearing three types of shoes | 20 automotive workers | Different types of safety shoes can influence the gait and distribution of plantar pressure. |
| Orr et al. (3) | Impact of footwear on workers | Systematic literature review | Systematic literature search following the PRISMA statement | 50 articles resulting from the literature search | Occupational footwear can impact on physical task performance and injury risk. |
| Sharifirad et al. (37) | Paddy field workers (Northern Iran, 2022) | Descriptive cross-sectional study | Standardized Nordic Questionnaire | 384 paddy field workers | There is a high prevalence of musculoskeletal problems among paddy field workers, particularly in the back, hips, knees, shoulders, and elbows. One of the potential risk factors may be footwear. |
| Wardle & Greeves (26) | Military personnel | Systematic literature review | Systematic literature search following the PRISMA statement | 61 articles resulting from the literature search | Modification of footwear and application of customised plantar orthoses with the aim of reducing musculoskeletal injuries in military personnel does not reduce their incidence and is not a recommended measure. |
| Schulze et al. (30) | Military personnel (Germany, 2014) | Descriptive cross-sectional study | Treadmill stride and video recording analysis, as well as goniometry measurement | 32 soldiers | Footwear has a relevant influence on gait parameters and functionality of the lower limb. Limitations of the ankle joint movement are influenced by the material and shape of the shoes. Rigid material may be the decisive factor, promoting the development of overloading syndromes such as shin splints. Future research should focus on the flexibility of the material used while ensuring functionality. |
| Sobhani et al. (29) | Military personnel (Iran, 2015) | Descriptive cross-sectional study | Measurement of anthropometric and anatomical criteria of randomly selected subjects | 181 male subjects | Military footwear (which was not standardised) had no influence on the incidence of medial tibial stress syndrome. |
| Paisis et al. (27) | Military personnel (Greece, 2013) | Descriptive cross-sectional study | Measurement of anthropometric and anatomical criteria of subjects | 7 young adults | There is little evidence on the use of insoles in military boots, so their use should be assessed on a case-by-case basis. |
| Nesterovica et al. (28) | Infantry soldiers (Latvia, 2021) | Descriptive cross-sectional study | Interview + Measurement of anthropometric and anatomical criteria of subjects | 227 soldiers | Improper boot size was significantly related to comfort ratings but was not associated with a history of lower leg overuse injury. |
| Garner et al. (32) | Firefighters (USA, 2012) | Descriptive cross-sectional study | To examine differences in worker stability with and without firefighting boots | 12 firefighters | The heavier the boots worn, the greater the fatigue and, consequently, the greater the alterations in balance and postural control in the worker. |
